# ID 255 - Tecnovigilância dos Dispositivos Médicos no Brasil nos Últimos 10 Anos.

**DOI:** 10.5327/2237-9622.2025.v34s1.255

**Published:** 2025-11-25

**Authors:** Cynthia Carolina Duarte Andrade, Natalliely Adriana da Luz, Patrícia Sousa Silva Pinto

**Keywords:** segurança do paciente, dispositivos médicos, eventos adversos, tecnovigilância

## Abstract

**Introdução:**

Os dispositivos médicos e equipamentos hospitalares estão em constante inovação e visam à qualidade e à efetividade nos diagnósticos e tratamentos dos pacientes. O monitoramento desses produtos de saúde é fundamental para garantir a segurança do paciente. A Agência Nacional de Vigilância Sanitária (Anvisa) tem um papel importante no controle de fabricação e fiscalização dos dispositivos para mitigar possíveis agravos decorrentes de sua utilização. Este estudo objetiva descrever os dados brasileiros de tecnovigilância dos dispositivos médicos no Brasil, de 2014 a 2024.

**Método:**

Consiste em um estudo descritivo sobre incidentes (eventos adversos) e queixas técnicas relacionados a dispositivos notificados no Brasil de 12 de setembro de 2015 a 12 de setembro de 2024. Os dados foram coletados do Power BI da Anvisa. Foram adotados os seguintes filtros: horizonte temporal de 12/9/2014 a 12/9/2024 e produto-motivo das notificações: artigos médicos. Para facilitar a compreensão dos dados, estes foram assim apresentados: frequências (absoluta e relativa) das notificações (eventos adversos e queixas técnicas); classificação destas quanto aos riscos associados ao uso dos dispositivos médicos; e identificação dos dispositivos médicos motivos, das não conformidades e dos eventos adversos (conforme a nomenclatura adotada pela Organização Mundial da Saúde – OMS) mais ocorrentes.

**Resultados:**

Nos últimos 10 anos, a Anvisa recebeu 149.099 notificações relacionadas a dispositivos médicos, sendo 23.716 (15,91%) referentes a eventos adversos e 125.383 (84,09%) relacionadas a queixas técnicas. Sobre os riscos associados ao uso dos dispositivos médicos, 57,24% (n=13.576) dos eventos adversos foram classificados como III (alto risco), e 45,35% (n=56,862) das queixas técnicas foram classificadas como II (médio risco). Os dispositivos médicos motivos mais ocorrentes nas notificações foram equipos, com 13.840 (9,28%); cateteres, com 11.834 (7,94%); implantes dentários, com 10.123 (6,79%); e seringas descartáveis, com 8.653 (5,80%). Entre as não conformidades informadas, quebras (n=7.762; 5,21%) e rachaduras (n=5.930; 3,98%) de materiais são as mais ocorrentes. Em apenas 15,45% das notificações, consta a identificação dos eventos adversos: falha e complicação de implante (n=10.708; 7,18%), intervenção cirúrgica (n=871; 0,58%), óbito (n=646; 0,43%), sangramento (n=522; 0,35%), dor (n=496; 0,33%) e lesão cutânea (n=423; 0,28%).

**Conclusão:**

Percebe-se que são mais frequentes as notificações de queixas técnicas que as de eventos adversos. Entende-se que, quanto mais utilizada a tecnologia, maior a chance de notificações reportadas à Anvisa, haja vista que, do total de notificações, 29,81% (n=44.450) referem-se a quatro dispositivos médicos motivos (equipos, cateteres, implantes dentários e seringas descartáveis) que são muito empregados nos diferentes serviços de saúde. Com relação às classes de risco dos dispositivos médicos, espera-se que, quanto mais invasiva uma tecnologia, maior o risco associado ao seu uso. Nesse sentido, a maior parte dos incidentes que causam danos ao paciente (eventos adversos) é da classe III (alto risco). Entre as possíveis dificuldades relacionadas à identificação dos eventos adversos, destacam-se a adoção da nomenclatura padronizada pela Organização Mundial da Saúde (OMS) e a investigação da relação causal entre o incidente e o dispositivo médico. O óbito, por exemplo, pode ser multicausal.

